# An Assessment of the Diversity of Culturable Bacteria from Main Root of Sugar Beet

**DOI:** 10.1264/jsme2.ME13182

**Published:** 2014-04-30

**Authors:** Kazuyuki Okazaki, Takao Iino, Yosuke Kuroda, Kazunori Taguchi, Hiroyuki Takahashi, Takuji Ohwada, Hiroto Tsurumaru, Takashi Okubo, Kiwamu Minamisawa, Seishi Ikeda

**Affiliations:** 1 Hokkaido Agricultural Research Center, National Agriculture and Food Research Organization, 9–4 Shinsei-minami, Memurochou, Kasai-gun, Hokkaido 082–0081, Japan; 2 Japan Collection of Microorganisms, RIKEN BioResource Center, 3–1–1 Koyadai, Tsukuba, Ibaraki 305–0074, Japan; 3 Department of Food Science, Obihiro University of Agriculture and Veterinary Medicine, Inada-cho, Obihiro, Hokkaido 080–8555, Japan; 4 Graduate School of Life Science, Tohoku University, 2–1–1 Katahira, Aoba-ku, Sendai, Miyagi 980–8577, Japan

**Keywords:** *Alphaproteobacteria*, endophyte, *Haloferula*, sugar beet root microbiome, *Verrucomicrobium*

## Abstract

The partial sequences of the 16S rRNA genes of 531 bacteria isolated from the main root of the sugar beet (*Beta vulgaris* L.) were determined and subsequently grouped into 155 operational taxonomic units by clustering analysis (≥99% identity). The most abundant phylum was *Proteobacteria* (72.5–77.2%), followed by *Actinobacteria* (9.8–16.6%) and *Bacteroidetes* (4.3– 15.4%). *Alphaproteobacteria* (46.7–64.8%) was the most dominant class within *Proteobacteria*. Four strains belonging to *Verrucomicrobia* were also isolated. Phylogenetic analysis revealed that the *Verrucomicrobia* bacterial strains were closely related to *Haloferula* or *Verrucomicrobium*.

The sugar beet (*Beta vulgaris* L.) is one of the world’s most important crops as a source of sucrose and has recently drawn attention as an energy plant (10). However, the increasing cost of chemicals for fertilizers and pesticides in recent years has become a serious problem for agricultural production. Although the biomass productivity of sugar beet is high, the mechanisms supporting the productivity of this plant remain unclear. The high biomass of the sugar beet may be attributed to its high affinity with beneficial microbes, as has also been reported in the sweet potato (18).

The diversity and community composition of culturable bacteria from the main root of the sugar beet were assessed in the present study using 16S rRNA gene sequencing in order to provide basic ecological information and construct a resource for an efficient survey and utilization of plant-growth-promoting rhizobacteria (PGPR).

The seeds of the sugar beet (cultivar “Rycka”) were sown on 27 April 2011 in an experimental field of the Hokkaido Agricultural Research Center (Memuro, Hokkaido, Japan, 42°89.2′ N/143°0.7.7′ E). The width of the rows and spacing between plants were 60 cm and 22.5 cm, respectively. The field was dressed with S014 (150, 315, and 210 kg/ha for N, P_2_O_5_, and K_2_O, respectively; Hokuren Fertilizer Co., Sapporo, Japan) as a basal fertilization. Based on a visual inspection, three healthy sugar beets were randomly sampled from the experimental field on 11 August 2011. The main roots were carefully washed with tap water to remove loosely adhering soil and debris and then with sterilized water. The lateral roots on the main root were manually removed, and the main root was then separated into surface (SU) and core (CO) parts. SU represented a part of the main root with a 10-mm thickness from the root surface, and CO represented a core part of the main root without the root surface. Approximately 200 g of each part of the tissues derived from three plants was added to 500 mL of 67 mM phosphate buffer (pH 7.0) and homogenized in a blender. The homogenate was filtered through a piece of Miracloth (Calbiochem, Darmstadt, Germany), and the supernatant was then stored as a 15% glycerol stock at –80°C until later bacterial isolation. Soil samples were collected from three sampling sites by an auger (between a depth of 5 cm to 15 cm) after removing surface soil on 12 October 2011, and were combined as a composite soil sample. The chemical characteristics of the soil sample were determined by the Tokachi Nokyoren Agricultural Research Institute (Obihiro, Hokkaido, Japan) (Table S1).

Four bacterial isolate collections were constructed from the SU and CO parts of the main root of the sugar beet by R2A (BD, Franklin Lakes, NJ, USA) and HM media. The HM medium was modified Cole’s HM medium (3) by adding 0.1% L-arabinose and 0.03% yeast extract. The pH was adjusted to 6.8 with 2N NaOH prior to autoclaving. Homogenates of the SU and CO parts of the main root were serially diluted with 67 mM phosphate buffer (pH 7.0), and 100 μl of each dilution was inoculated on 1.5% agar plates of R2A medium containing 50 mg L^−1^ cycloheximide and HM medium containing 50 mg L^−1^ polymyxin B. After an incubation at 24°C in the dark for 7 d, colonies were randomly collected and subjected to single colony isolation twice by streaking them onto fresh medium. The purified bacterial strains were stored as a 15% glycerol stock at –25°C.

Regarding DNA extraction, strains were cultured on an agar plate of the R2A or HM medium for a few days at 24°C. An aliquot of bacterial cells was collected with an inoculation loop and total DNA was extracted from the cells using a previously described DNA extraction method (7). PCR amplification for 16S rRNA gene sequencing, and the editing and analyses of sequences for the strains isolated in the present study were conducted as previously described (17).

A total of 531 strains were isolated from the surface and core parts of the main root of the sugar beet using two media (Table 1). Clustering analysis (≥99% identity) was used to group 531 strains into 155 OTUs, and library coverage was 83.1%. Statistical analysis revealed that the number of OTUs and both Shannon and Simpson diversity indexes were higher for the surface tissue collection than for the core tissue collection in R2A and HM media (Table 1). However, these differences were small, which was consistent with the findings reported by Lilley *et al.* (12). Differences were also attributed to the thickness of the surface tissue (10 mm), which may have led to a large physical overlap between the surface and core samples.

*Proteobacteria* was the most dominant phylum in all isolate collections (72.5–77.2%) (Table 2). In contrast to previous studies in which the dominancy of *Gammaproteobacteria*, *Actinobacteria*, and *Firmicutes* was reported (8, 11, 12, 16), *Alphaproteobacteria* was the most abundant in all the collections in the present study (46.7–64.8%). These differences between the present and previous studies may be due to variations in the soil, fertilization conditions, or isolation methods. At the lower taxonomic ranks, three genera (*Bosea*, *Devosia*, and *Mesorhizobium*) in the order *Rhizobiales* were found to be stably present in all collections as the dominant genus (Table 2). Clustering analysis revealed that four genera (*Devosia*, *Mesorhizobium*, *Rhizobium*, and *Sphingomonas*) were highly diverse at the species level (Fig. S1). In *Alphaproteobacteria*, more than half of the OTUs (32 out of 63 OTUs) belonged to these four genera, which indicated a high micro-diversity.

*Actinobacteria* was the secondary dominant phylum following *Proteobacteria* in all collections (9.8–16.6%) (Table 2). *Microbacterium* and *Mycobacterium* were stably detected in all collections (Table 2 and Table S2). *Bacteroidetes* was more abundant in collections with the HM medium than in collections with the R2A medium (Table 2). Clustering analysis revealed a high microdiversity at the species level for both *Actinobacteria* and *Bacteroidetes* (28 and 27 OTUs in a total 155 OTUs, respectively) (Fig. S1).

Four strains belonging to *Verrucomicrobia* were isolated from the surface part of the main root of the sugar beet using the R2A medium. These strains were closely related to two genera, *Verrucomicrobium* and *Haloferula* (Fig. 1). The phylum *Verrucomicrobia* is known to be nearly ubiquitous and highly abundant in soils (1), but is generally considered to be recalcitrant for cultivation (4). To date, only three studies have reported the successful cultivation of *Verrucomicrobia* bacteria from a rhizosphere (5, 6, 13). The members of this phylum have been phylogenetically classified into 7 subdivisions (5). Phylogenetic tree analysis of the partial 16S rRNA gene sequences revealed that all four strains of *Verrucomicrobia* bacteria isolated in the present study belonged to subdivision 1 (Fig. 1). *Verrucomicrobia* bacteria belonging to subdivision 1 were also isolated from a rhizosphere in previous studies. The BvORR071 and BvORR085 strains isolated in the present study were closely related to the genus *Haloferula*. In this genus, *Haloferula luteola* was previously identified as a root endophyte of a halophyte (2). The results of the present as well as the findings of previous studies suggest that subdivision 1 is an important community member for a rhizosphere in diverse plants. Understanding the functional roles of *Verrucomicrobia* bacteria in adapting to a rhizosphere, which is a relatively high nutrient environment, is of importance because *Verrucomicrobia* is generally considered to be an oligotroph adapting to a low nutrient environment (5).

The HM medium used is a synthetic medium that has empirically and frequently been used to cultivate *Bradyrhizobium* strains with the addition of polymyxin B (9, 15). *Bradyrhizobium betae* was previously isolated from the main root of the sugar beet (14); therefore, it was assumed that the HM medium can be used for the preferential isolation of *Bradyrhizobium* sp. from the main root of the sugar beet. However, the isolation of *Bradyrhizobium* sp. strains using the HM medium was rare in the present study. Instead, *Alphaproteobacteria*, *Betaproteobacteria*, and *Bacteroidetes* were exclusively isolated using this medium. A high microdiversity was observed in these taxa (Fig. S1). These characteristics of the HM medium could be considered as an advantage for the efficient isolation for *Alphaproteobacteria*, *Betaproteobacteria*, and *Bacteroidetes* from an environment.

In conclusion, the present study revealed a high diversity in the culturable bacterial community, especially for *Alphaproteobacteria*, from the main root of the sugar beet. *Verrucomicrobia* bacteria belonging to two genera, *Verrucomicrobium* and *Haloferula*, were obtained using a standard R2A medium. Functional analyses of these sugar beet-associated bacteria should be conducted in future studies in order to clarify their ecological roles in a rhizosphere.

The nucleotide sequences were deposited in the DDBJ/EMBL/GenBank database. The sequence data for main root-associated bacteria isolated from SU with R2A and HM media were deposited under the accession numbers of AB851230–AB851416 and AB851138–AB851229, respectively. The sequence data from CO with R2A and HM media were deposited under the accession numbers of AB850977–AB851137 and AB850886–AB850976, respectively. *Haloferula* spp. BvORR071 and BvORR085 and *Verrucomicrobium* sp. BvORR034 were deposited to the Japan Collection of Microorganisms at the RIKEN Bioresource Center (RIKEN-BRC JCM) under the culture collection accession numbers JCM 18780, JCM 18781, and JCM 18782, respectively.

## Supplementary Information



## Figures and Tables

**Fig. 1:**
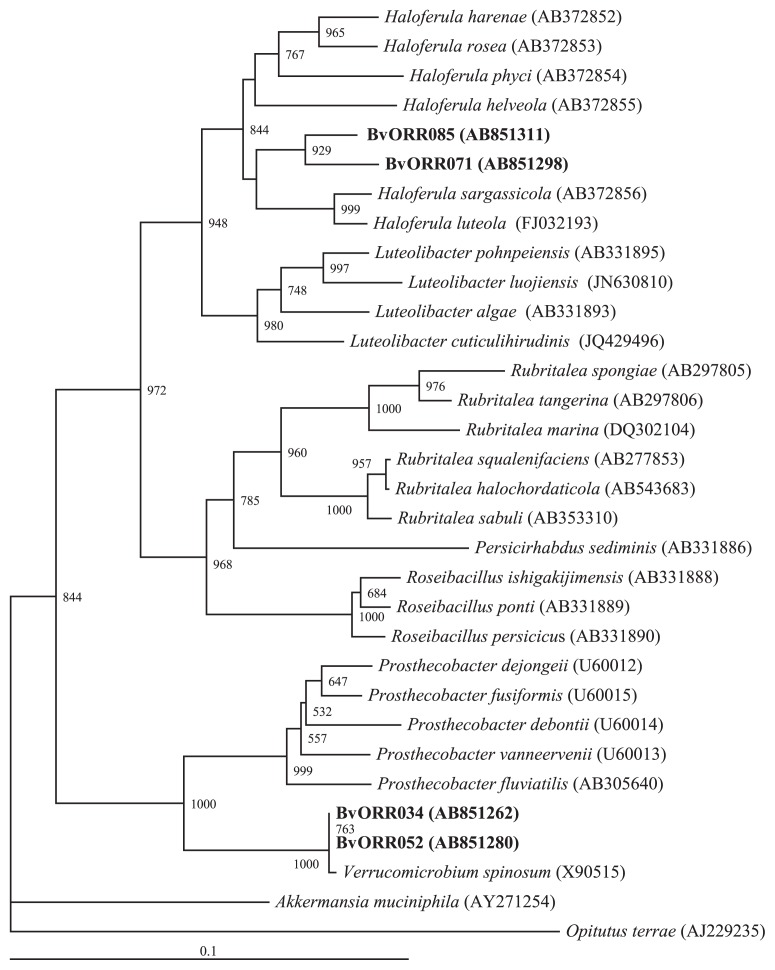
Phylogenetic tree analysis of 16S rRNA gene sequences of *Verrucomicrobia* strains isolated from the main root of the sugar beet. The tree was constructed by the neighbor-joining method with the reference sequences in subdivision 1 of *Verrucomicrobia* and the strains of *Verrucomicrobia* bacteria isolated in the present study (BvORR085, BvORR071, BvORR034, and BvORR052). The strains isolated in the present study were indicated in a bold font. The accession numbers are given in parentheses. *Opitutus terrae* was used as an out group. The scale represents 0.1 substitutions per site. The numbers at the nodes are the proportions of 1,000 bootstrap resamplings, and values above 500 are shown.

**Table 1:** Statistical characteristics of bacterial isolate collections derived from main roots of sugar beet

	Bacterial isolate collections				
		
Media	R2A		HM		Total
			
Tissue types	SU^a^	CO^a^	SU	CO	
Statistics					
No. of sequences	187	161	92	91	531
No. of OTUs^b^	78	67	52	40	155
No. of singletons	43	44	33	24	90
Library coverage (%)^c^	77.0	72.7	64.1	73.6	83.1
Diversity indexes					
Chao1	131.1	303.5	92.6	67.6	
ACE	194.1	273.2	159.1	124.8	
Shannon index (*H′*)	4.0	3.8	3.7	3.2	
Simpson index (1/*D*)	43.3	40.9	47.0	17.3	

a SU and CO stand for the surface and core portions of main roots of sugar beet, respectively.

b OTUs (Operational taxonomic units) were defined at 99% sequence identity by using MOTHUR.

c C=1– (*n1/N*), where *n1* is the number of singletons that appear once in a library, and *N* is the total number of strains in a isolate collection.

**Table 2:** Phylogenetic compositions of bacterial isolate collections derived from main roots of sugar beet

	Bacterial isolate collections (%)^a^			
	
Media	R2A		HM	
		
Tissue types	SU^b^	CO^b^	SU	CO
Proteobacteria^c^	77.0	75.2	77.2	72.5
Alphaproteobacteria	48.1	52.2	46.7	64.8*
*Asticcacaulis*	—	—	1.1	—
*Bosea*	5.3	5.6	6.5	11.0
*Caulobacter*	2.7	0.6	4.3	3.3
*Devosia*	9.1	12.4	6.5	29.7**††
*Labrys*	2.1	4.3	—	—
*Mesorhizobium*	8.0	5.0	4.3	6.6
*Rhizobium*	3.2	1.2	6.5	1.1
*Sphingomonas*	3.2	5.0	2.2	3.3
*Sphingopyxis*	4.3	4.3	1.1	—
unclassified				
Bradyrhizobiaceae	2.7	7.5	—	3.3
Others	7.5	6.2	14.1	6.5
Betaproteobacteria	9.6	6.8	20.7†	7.7*
*Polaromonas*	5.9	1.9	9.8	3.3
*Variovorax*	0.5	3.1	7.6	3.3††
Others	3.2	1.8	3.3	1.1
Gammaproteobacteria	18.2	16.1	5.4††	—*††
*Lysobacter*	5.9	6.2	3.3	—
*Pseudoxanthomonas*	9.1	6.8	—††	0.0††
Others	3.2	3.1	2.1	—
Others	1.1	—	4.3	—
Actinobacteria	16.6	15.5	9.8	12.1
*Aeromicrobium*	3.2	3.1	—	—
*Marmoricola*	0.5	—	—	—
*Microbacterium*	2.1	3.1	4.3	2.2
*Mycobacterium*	4.3	2.5	2.2	6.6
*Nocardioides*	4.8	1.2	—†	—
Others	1.7	5.6	3.3	3.3
Bacteroidetes	4.3	5.6	13.0††	15.4††
*Dyadobacter*	1.1	1.2	2.2	7.7†
*Mucilaginibacter*	—	—	4.3††	1.1
*Pedobacter*	1.6	1.9	3.3	2.2
Others	1.6	2.5	3.2	4.4
Firmicutes	—	3.7**	—	—
Verrucomicrobia	2.1	—	—	—

a The relative abundance in a bacterial isolate collection is shown in percentage.

b SU and CO stand for the surface and core portions of main roots of sugar beet, respectively.

c Sequences were classified using the Classifier of Ribosomal Database Project II release 10 with a confidence threshold 80%.

*, ** indicate statistical significance examined by the Library Compare of Ribosomal Database Project II at *p*<0.05 and *p*<0.01, respectively, between SU and CO in each medium.

†, †† indicate statistical significance examined by the Library Compare of Ribosomal Database Project II at *p*<0.05 and *p*<0.01, respectively, between R2A and HM in each of SU and CO.
