# Supplementary material for: An Assessment of the Diversity of Culturable Bacteria from Main Root of Sugar Beet
Source: Microbes Environ. 2014 Apr 30;29(2):220–3. doi: 10.1264/jsme2.ME13182 (PMC4103529; doi:10.1264/jsme2.ME13182)
Supplement: Supplementary file 1 [file 29_220_s1.pdf]

Supplementary Materials

An Assessment of the Diversity of Culturable Bacteria Associated with Main Roots of Sugar Beet

Kazuyuki Okazaki (1), Takao Iino (2), Yosuke Kuroda (1), Kazunori Taguchi (1), Hiroyuki Takahashi (1) , Takuji Ohwada (3), Hiroto Tsurumaru (4), Takashi Okubo (4), Kiwamu Minamisawa (4), and Seishi Ikeda (1)*

(1) Hokkaido Agricultural Research Center, National Agriculture and Food Research Organization, 9-4 Shinsei-minami, Memuro-chou, Kasai-gun, Hokkaido 082-0081, Japan; (2) Japan Collection of Microorganisms, RIKEN BioResource Center, 3-1-1 Koyadai, Tsukuba, Ibaraki 305-0074, Japan; (3) Department of Food Science, Obihiro University of Agriculture and Veterinary Medicine, Inada-cho, Obihiro, Hokkaido 080-8555, Japan; (4) Graduate School of Life Science, Tohoku University, 2-1-1 Katahira, Aoba-ku, Sendai, Miyagi 980-8577, Japan

Running Title: Main root-associated bacteria in sugar beet

*Corresponding author: Seishi Ikeda, Seishi Ikeda, Hokkaido Agricultural Research Center, National Agricultural and Food Research Organization, 9-4 Shinsei-minami, Memuro-chou, Kasai-gun, Hokkaido 082-0081, Japan. E-mail: sikeda67@affrc.go.jp; Tel: +81-155-62-9276; Fax: +81-155-61-2127.

Table S1. Characteristics of field soil used in the present study

pH (H_2O)	Humid content (%)	Total nitrogen (%)	NO_3-N (mg/100g)	NH_4-N (mg/100g)	P_2O_5 (mg/100g)	Phosphate absorption coefficient	K_2O (mg/100g)	CaO (mg/100g)	MgO (mg/100g)	CEC (me/100g)
6.3	6.2	0.32	0.55	0.34	16.5	1786	39.9	441.8	51.1	31.9

Table S2. Phylogenetic compositions of bacterial isolate collections derived from main roots of sugar beet

Taxa (%)[a]	Bacterial isolate collections			
	R2A		HM	
	SU[b]	CO[b]	SU	CO
Proteobacteria	77.0	75.2	77.2	72.5
Alphaproteobacteria	48.1	52.2	46.7	64.8*
Rhodospirillales	0.0	0.0	1.1	1.1
Rhodospirillaceae	0.0	0.0	1.1	1.1
Inquilinus	0.0	0.0	1.1	1.1
Sphingomonadales	9.6	0.0	9.8	0.0
Sphingomonadaceae	9.1	11.2	9.8	5.5
Sphingobium	0.5	0.6	2.2	0.0
Sphingopyxis	4.3	4.3	1.1	0.0
Novosphingobium	1.1	1.2	4.3	2.2
Sphingomonas	3.2	5.0	2.2	3.3
Erythrobacteraceae	0.5	0.0	0.0	0.0
unclassified Erythrobacteraceae	0.5	0.0	0.0	0.0
Caulobacterales	3.7	1.2	6.5	3.3
Caulobacteraceae	3.7	1.2	6.5	3.3
Brevundimonas	1.1	0.6	1.1	0.0
Caulobacter	2.7	0.6	4.3	3.3
Asticcacaulis	0.0	0.0	1.1	0.0
Rhizobiales	34.8	39.8	28.3	54.9***†
Beijerinckiaceae	0.5	0.6	0.0	0.0
Beijerinckia	0.5	0.6	0.0	0.0
Xanthobacteraceae	2.1	4.3	0.0	0.0
Labrys	2.1	4.3	0.0	0.0
Rhizobiaceae	3.2	1.2	6.5	1.1
Rhizobium	3.2	1.2	6.5	1.1
Bradyrhizobiaceae	9.6	14.9	6.5	15.4
Afipia	0.0	0.6	0.0	0.0
Bradyrhizobium	1.6	1.2	0.0	1.1
Bosea	5.3	5.6	6.5	11.0
unclassified Bradyrhizobiaceae	2.7	7.5	0.0	3.3
Phyllobacteriaceae	9.1	6.2	5.4	7.7
Phyllobacterium	0.0	0.6	0.0	0.0
Aminobacter	0.0	0.6	0.0	1.1
Mesorhizobium	8.0	5.0	4.3	6.6
unclassified Phyllobacteriaceae	1.1	0.0	1.1	0.0
Hyphomicrobiaceae	9.1	12.4	6.5	29.7**††
Devosia	9.1	12.4	6.5	29.7**††
Methylobacteriaceae	0.5	0.0	1.1	0.0
Methylobacterium	0.5	0.0	1.1	0.0
unclassified Rhizobiales	0.5	0.0	2.2	1.1
unclassified Alphaproteobacteria	0.0	0.0	1.1	0.0

Table S2. Continued

Taxa (%)[a]	Bacterial isolate collections			
	R2A		HM	
	SU[b]	CO[b]	SU	CO
Betaproteobacteria	9.6	6.8	20.7[†]	7.7[*]
Burkholderiales	9.6	6.8	20.7[†]	7.7[*]
Burkholderiales_incertae_sedis	0.0	0.6	0.0	0.0
Aquabacterium	0.0	0.6	0.0	0.0
Burkholderiaceae	0.0	0.0	1.1	0.0
Burkholderia	0.0	0.0	1.1	0.0
Oxalobacteraceae	0.5	0.6	1.1	0.0
Herminiimonas	0.5	0.6	0.0	0.0
Herbaspirillum	0.0	0.0	1.1	0.0
Comamonadaceae	9.1	5.6	18.5[†]	7.7[*]
Polaromonas	5.9	1.9	9.8	3.3
Roseateles	0.0	0.0	0.0	1.1
Variovorax	0.5	3.1	7.6[††]	3.3
Acidovorax	1.1	0.0	0.0	0.0
Pelomonas	0.5	0.0	0.0	0.0
Curvibacter	0.5	0.0	0.0	0.0
unclassified Comamonadaceae	0.5	0.6	1.1	0.0
Gammaproteobacteria	18.2	16.1	5.4[††]	0.0[*††]
Pseudomonadales	0.5	0.6	1.1	0.0
Pseudomonadaceae	0.5	0.6	1.1	0.0
Rhizobacter	0.5	0.6	0.0	0.0
Pseudomonas	0.0	0.0	1.1	0.0
Xanthomonadales	17.6	15.5	4.3[††]	0.0[††]
Xanthomonadaceae	17.6	15.5	4.3[††]	0.0[††]
Rhodanobacte	0.5	0.6	1.1	0.0
Lysobacter	5.9	6.2	3.3	0.0[†]
Dokdonella	1.6	1.9	0.0	0.0
Pseudoxanthomonas	9.1	6.8	0.0[††]	0.0[††]
Stenotrophomonas	0.5	0.0	0.0	0.0
unclassified Proteobacteria	1.1	0.0	4.3	0.0

Table S2. Continued

Taxa (%)[a]	Bacterial isolate collections			
	R2A		HM	
	SU[b]	CO[b]	SU	CO
Actinobacteria	16.6	15.5	9.8	12.1
Actinobacteria	16.6	15.5	9.8	12.1
Actinomycetales	16.6	15.5	9.8	12.1
Streptomycetaceae	0.5	1.2	2.2	0.0
Streptomyces	0.5	1.2	2.2	0.0
Nocardioidaceae	9.6	5.6	0.0[††]	0.0[†]
Nocardioides	4.8	1.2	0.0[†]	0.0
Aeromicrobium	3.2	3.1	0.0	0.0
Kribbella	0.5	0.0	0.0	0.0
Marmoricola	0.5	0.0	0.0	0.0
unclassified Nocardioidaceae	0.5	1.2	0.0	0.0
Microbacteriaceae	2.1	6.2	4.3	4.4
Pseudoclavibacter	0.0	0.0	0.0	1.1
Leifsonia	0.0	0.6	0.0	1.1
Microbacterium	2.1	3.1	4.3	2.2
Salinibacterium	0.0	0.6	0.0	0.0
Agrococcus	0.0	1.9	0.0	0.0
Nocardiaceae	0.0	0.0	0.0	1.1
Nocardia	0.0	0.0	0.0	1.1
Mycobacteriaceae	4.3	2.5	2.2	6.6
Mycobacterium	4.3	2.5	2.2	6.6
Pseudonocardiaceae	0.0	0.0	1.1	0.0
Amycolatopsis	0.0	0.0	1.1	0.0
Bacteroidetes	4.3	5.6	13.0[††]	15.4[††]
Flavobacteria	0.0	0.6	0.0	1.1
Flavobacteriales	0.0	0.6	0.0	1.1
Flavobacteriaceae	0.0	0.6	0.0	1.1
Flavobacterium	0.0	0.6	0.0	1.1
Sphingobacteria	4.3	5.0	13.0[††]	14.3[†]
Sphingobacteriales	4.3	5.0	13.0[††]	14.3[†]
Chitinophagaceae	1.6	1.2	3.3	3.3
Sediminibacterium	0.0	0.6	0.0	0.0
Chitinophaga	0.0	0.0	1.1	1.1
Niabella	0.5	0.0	0.0	1.1
Lacibacter	0.5	0.0	1.1	0.0
Niastella	0.0	0.0	1.1	0.0
unclassified Chitinophagaceae	0.5	0.6	0.0	1.1
Sphingobacteriaceae	1.6	2.5	7.6[†]	3.3
Sphingobacterium	0.0	0.6	0.0	0.0
Mucilaginibacter	0.0	0.0	4.3[††]	1.1
Pedobacter	1.6	1.9	3.3	2.2
Cytophagaceae	1.1	1.2	2.2	7.7[††]
Dyadobacter	1.1	1.2	2.2	7.7[††]

Table S2. Continued

Taxa (%)[a]	Bacterial isolate collections			
	R2A		HM	
	SU[b]	CO[b]	SU	CO
Firmicutes	0.0	3.7^{**}	0.0	0.0
Bacilli	0.0	3.7^{**}	0.0	0.0
Bacillales	0.0	3.7^{**}	0.0	0.0
Bacillaceae 1	0.0	1.9	0.0	0.0
Bacillus	0.0	1.9	0.0	0.0
Paenibacillaceae	0.0	1.9	0.0	0.0
Paenibacillus	0.0	1.9	0.0	0.0
Verrucomicrobia	2.1	0.0	0.0	0.0
Verrucomicrobiae	2.1	0.0	0.0	0.0
Verrucomicrobiales	2.1	0.0	0.0	0.0
Verrucomicrobiaceae	2.1	0.0	0.0	0.0
Haloferula	1.1	0.0	0.0	0.0
Verrucomicrobium	1.1	0.0	0.0	0.0

[a]Sequences were classified using the Classifier of Ribosomal Database Project II release 10 with a confidence threshold 80%.

[b]SUand CO stand for the surface and core parts of main roots of sugar beet, respectively.

*, ** indicate statistical significance examined by the Library Compare of Ribosomal Database Project II at p <0.05 and p <0.01, respectively, between SU and CO in each medium.

†, †† indicate statistical significance examined by the Library Compare of Ribosomal Database Project II at p<0.05 and p<0.01, respectively, between R2A and HM in each part of main roots.

1

Figure legends

Fig. S1. Phylogenic distribution of OTUs at species level for main root-associated bacteria in sugar beet. OTUs (Operational taxonomic units) were defined at 99% sequence identity by using MOTHUR. *Aquifex pyrophilus* (M83548) was used as an out group for the dendrogram. OTUs were expressed as prefixes derived from phylum identity (Symbols; AP, *Alphaproteobacteria*; BP, *Betaproteobacteria*; GP, *Gammaproteobacteria*; B, *Bacteroidetes*; F, *Firmicutes*; A, *Actinobacteria*; V, *Verrucomicrobia*, with consecutive numbers). The table indicates the relative abundance (%) of isolates belonging to each OTU in each collection and the result of BLAST search using representative sequence. SU and CO stand for the surface and core parts of main roots of sugar beet, respectively. *, ** indicate statistical significance examined by Fisher's exact test at p<0.05 and p<0.01, respectively, between SU and CO in each medium. †, †† indicate statistical significance examined by Fisher's exact test at p<0.05 and p<0.01, respectively, between R2A and HM in each of SU and CO.

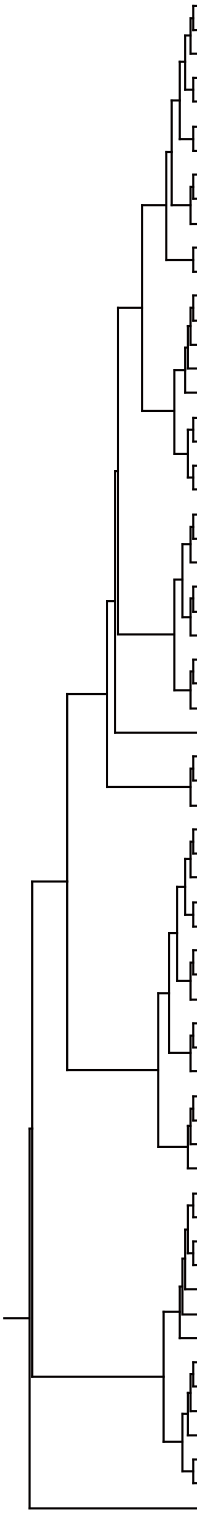

OTUs	Isolate collections (%)				Closest relatives in known species	Acc. No.	Identity (%)
	R2A		HM				
	SU	CO	SU	CO			
AP1	-	-	-	1.1	*Mesorhizobium loti*	FN386773	98
AP2	-	-	1.1	-	*Mesorhizobium ciceri*	NR 074211	99
AP3	3.2	-	-*	-	*Mesorhizobium loti*	FN386773	100
AP4	1.6	2.5	1.1	-	*Mesorhizobium* sp.	JX292574	100
AP5	2.7	2.5	2.2	5.5	*Mesorhizobium amorphae*	AB741445	100
AP6	0.5	-	-	-	*Mesorhizobium albiziae*	NR 043549	98
AP7	0.5	-	-	-	*Mesorhizobium* sp.	FJ544261	98
AP8	-	0.6	-	-	*Aminobacter aminovorans*	EF473294	95
AP9	-	-	1.1	-	*Aminobacter aminovorans*	AM285009	95
AP10	-	-	-	1.1	*Aminobacter aminovorans*	EF473294	100
AP11	-	0.6	-	-	*Phyllobacterium myrsinacearum*	EU420076	98
AP12	0.5	-	-	-	*Phyllobacterium myrsinacearum*	DQ370018	100
AP13	-	-	-	1.1	*Rhizobium radiobacter*	KC196495	99
AP14	0.5	-	-	-	*Rhizobium radiobacter*	GU826585	99
AP15	-	0.6	-	-	*Rhizobium radiobacter*	JX971560	100
AP16	1.1	0.6	5.4†	-	*Rhizobium galegae*	Y12360	100
AP17	0.5	-	-	-	*Rhizobium daejeonense*	AB681831	99
AP18	-	-	1.1	-	*Rhizobium sullae*	FJ785219	100
AP19	0.5	-	-	-	*Rhizobium petrolearium*	EU556969	98
AP20	-	-	1.1	-	*Rhizobium giardinii*	HQ263115	97
AP21	0.5	-	-	-	*Rhizobium giardinii*	HQ263115	99
AP22	-	-	-	1.1	*Devosia insulae*	NR 044036	97
AP23	4.3	3.7	1.1*	7.7	*Devosia insulae*	NR 044036	99
AP24	0.5	-	2.2	-	*Devosia ginsengisoli*	KC676321	100
AP25	-	-	1.1	-	*Devosia insulae*	NR 044036	96
AP26	1.6	3.1	-	2.2	*Devosia insulae*	NR 044036	99
AP27	0.5	-	-	-	*Devosia insulae*	NR 044036	98
AP28	1.6	5.0	2.2**	19.8††	*Devosia neptuniae*	JX841065	100
AP29	1.1	-	2.2	-	*Devosia limi*	NR 042324	99
AP30	-	0.6	-	-	*Devosia subaequoris*	NR 042544	98
AP31	0.5	0.6	-	-	*Beijerinckia derxii*	NR 042182	96
AP32	-	0.6	-	-	*Labrys methylaminiphilus*	NR 041336	99
AP33	1.1	3.7	-	-	*Labrys methylaminiphilus*	NR 041336	100
AP34	1.1	-	-	-	*Labrys methylaminiphilus*	NR 041336	98
AP35	-	0.6	-	1.1	*Bradyrhizobium elkanii*	KC787046	99
AP36	-	0.6	-	-	*Afipia felis*	AY548800	100
AP37	0.5	-	-	-	*Afipia birgiae*	NR 025117	99
AP38	0.5	1.9	-	-	*Bradyrhizobium japonicum*	AB614498	100
AP39	1.1	-	-	-	*Bradyrhizobium japonicum*	JN558795	99
AP40	-	0.6	-	1.1	*Bradyrhizobium japonicum*	KC736656	98
AP41	0.5	-	-	-	*Bradyrhizobium elkanii*	HQ233241	98
AP42	1.6	5.6	-	2.2	*Rhodopseudomonas rhenobacensis*	FN796846	98
AP43	0.5	0.6	1.1	2.2	*Bosea thiooxidans*	DQ104985	99
AP44	4.8	5.0	5.4	8.8	*Bosea thiooxidans*	JN627995	100
AP45	0.5	-	1.1	-	*Methylobacterium adhaesivum*	AB698724	100
AP46	-	-	-	1.1	*Caulobacter segnis*	NR 074208	99
AP47	2.7	0.6	4.3	2.2	*Caulobacter henricii*	NR 025319	99
AP48	1.1	0.6	1.1	-	*Brevundimonas vesicularis*	KC494327	100
AP49	-	-	1.1	-	*Asticcacaulis biprosthecium*	AJ247193	98
AP50	-	-	-	1.1	*Sphingomonas mali*	JN592470	98
AP51	1.1	0.6	2.2	-	*Sphingomonas mali*	AB680885	98
AP52	-	0.6	-	-	*Sphingomonas asaccharolytica*	AB680883	100
AP53	2.1	1.9	-	-	*Sphingomonas oligophenolica*	NR 024685	99
AP54	-	-	1.1	-	*Sphingomonas pituitosa*	JF459949	91
AP55	-	1.9	-	2.2	*Sphingomonas azotifigens*	JQ579646	98
AP56	0.5	0.6	2.2	-	*Sphingobium aromaticiconvertens*	NR 042479	98
AP57	-	0.6	-	-	*Novosphingobium hassiacum*	NR 028962	99
AP58	0.5	0.6	4.3†	2.2	*Novosphingobium subterraneum*	JF459977	98
AP59	0.5	-	-	-	*Novosphingobium naphthalenivorans*	AB649005	99
AP60	0.5	-	-	-	*Erythrobacter flavus*	KC583223	98
AP61	3.2	4.3	1.1	-	*Sphingopyxis panaciterrae*	AB245354	99
AP62	1.1	-	-	-	*Sphingopyxis witflariensis*	NR 028010	99
AP63	-	-	1.1	1.1	*Inquilinus ginsengisoli*	AB245352	100

Fig. S1 K. Okazaki

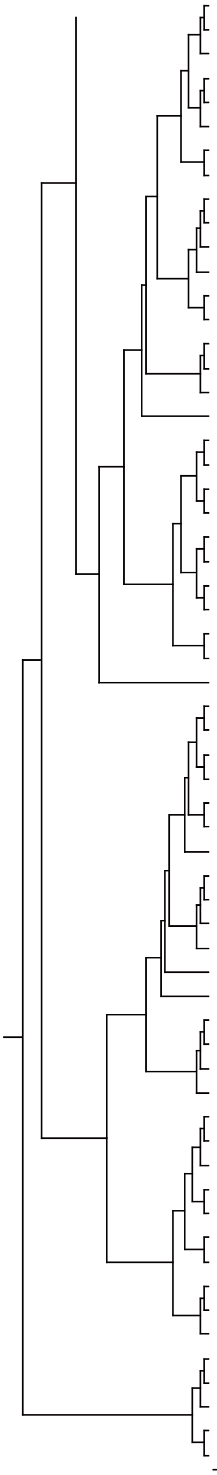

OTUs	Isolate collections (%)				Closest relatives in known species	Acc. No.	Identity (%)
	R2A		HM				
	SU	CO	SU	CO			
BP1	-	-	-	1.1	*Polaromonas ginsengisoli*	AB245355	99
BP2	5.9	1.9	7.6	2.2	*Polaromonas ginsengisoli*	AB245355	100
BP3	-	-	2.2	-	*Polaromonas aquatica*	NR 042404	99
BP4	-	-	1.1	-	*Curvibacter delicatus*	AB680705	97
BP5	0.5	-	-	-	*Curvibacter delicatus*	NR 028713	98
BP6	0.5	0.6	-	-	*Albidiferax ferrireducens*	AM265401	98
BP7	0.5	3.1	7.6††	3.3	*Variovorax paradoxus*	NR 074646	100
BP8	1.1	-	-	-	*Acidovorax radicis*	JF915365	100
BP9	-	-	1.1	-	*Rhizobacter fulvus*	AB649013	98
BP10	0.5	0.6	-	-	*Rhizobacter fulvus*	AB649013	99
BP11	1.1	-	2.2	-	*Rhizobacter fulvus*	AB649013	97
BP12	-	0.6	-	-	*Aquabacterium parvum*	NR 024874	98
BP13	-	-	-	1.1	*Mitsuaria chitosanitabida*	FJ796447	99
BP14	0.5	-	-	-	*Pelomonas soli*	EF660749	99
BP15	-	0.6	-	-	*Herminiimonas fonticola*	AB512142	99
BP16	0.5	-	-	-	*Herminiimonas saxobsidens*	NR 042610	100
BP17	-	-	1.1	-	*Herbaspirillum hiltneri*	DQ150563	100
BP18	-	-	1.1	-	*Burkholderia soli*	NR 043872	99
GP1	-	0.6	-	-	*Dokdonella fugitiva*	NR 042397	97
GP2	0.5	0.6	-	-	*Dokdonella fugitiva*	NR 042397	98
GP3	0.5	0.6	-	-	*Dokdonella koreensis*	EF589679	95
GP4	0.5	-	-	-	*Dokdonella koreensis*	NR 043322	96
GP5	9.1	6.8	-††	-††	*Pseudoxanthomonas ginsengisoli*	AB245360	100
GP6	0.5	-	-	-	*Stenotrophomonas rhizophila*	KC790309	100
GP7	4.3	-	1.1**	-	*Lysobacter niastensis*	JQ342848	100
GP8	1.6	6.2	2.2*	-†	*Lysobacter gummosus*	NR 041005	99
GP9	-	0.6	1.1	-	*Rhodanobacter ginsenosidimutans*	AB682427	98
GP10	0.5	-	-	-	*Rhodanobacter thiooxydans*	AB741464	100
GP11	-	-	1.1	-	*Pseudomonas extremorientalis*	NR 025174	100
B1	-	-	1.1	-	*Pedobacter terrae*	GU385862	99
B2	-	-	1.1	-	*Pedobacter kribbensis*	EF660752	100
B3	0.5	-	1.1	-	*Pedobacter borealis*	NR 044381	99
B4	0.5	-	-	1.1	*Pedobacter agri*	JX122157	98
B5	-	0.6	-	1.1	*Pedobacter panaciterrae*	EF195087	100
B6	0.5	-	-	-	*Pedobacter africanus*	NR 028977	99
B7	-	1.2	-	-	*Pedobacter koreensis*	NR 043538	99
B8	-	-	2.2	-	*Mucilaginibacter dorajii*	GU139697	95
B9	-	-	1.1	-	*Mucilaginibacter kameinonensis*	AB681894	98
B10	-	-	1.1	-	*Mucilaginibacter paludis*	NR 042597	97
B11	-	-	-	1.1	*Mucilaginibacter dorajii*	GU139697	94
B12	-	0.6	-	-	*Sphingobacterium composti*	JF731241	93
B13	-	0.6	-	1.1	*Flavobacterium johnsoniae*	AM921621	97
B14	-	-	-	3.3†	*Dyadobacter fermentans*	NR 074368	98
B15	1.1	0.6	1.1	2.2	*Dyadobacter fermentans*	DQ922762	100
B16	-	0.6	-	2.2	*Dyadobacter koreensis*	AB681383	97
B17	-	-	1.1	-	*Dyadobacter ginsengisoli*	NR 041372	95
B18	-	-	-	1.1	*Niastella populi*	EU877262	94
B19	0.5	-	-	-	*Niastella populi*	EU877262	93
B20	-	-	1.1	-	*Niastella populi*	EU877262	94
B21	0.5	-	-	1.1	*Niabella yanshanensis*	FJ457040	98
B22	-	0.6	-	-	*Sediminibacterium salmoneum*	NR 044197	93
B23	-	-	-	1.1	*Chitinophaga g insengisegetis*	NR 041486	99
B24	-	-	1.1	-	*Chitinophaga g insengisegetis*	NR 041486	99
B25	-	-	1.1	-	*Chitinophaga n iabensis*	AB682428	86
B26	0.5	-	-	-	*Chitinophaga n iabensis*	NR 044559	87
B27	-	0.6	-	-	*Chitinophaga filiformis*	JQ659521	88
F1	-	0.6	-	-	*Paenibacillus cineris*	EU839659	99
F2	-	0.6	-	-	*Paenibacillus amylolyticus*	AM237394	100
F3	-	0.6	-	-	*Paenibacillus alginolyticus*	JX464208	98
F4	-	0.6	-	-	*Bacillus ginsengihumi*	HQ219843	100
F5	-	1.2	-	-	*Bacillus arbutinivorans*	KC315762	100

Fig. S1 Continued K. Okazaki

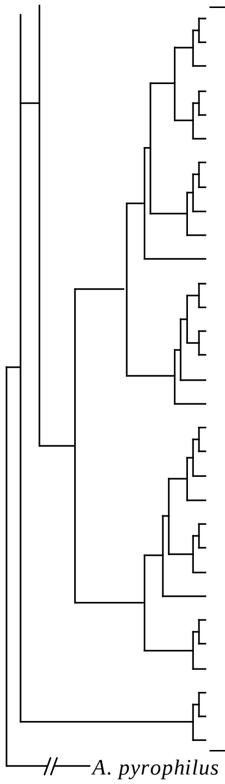

OTUs	Isolate collections (%)				Closest relatives in known species	Acc. No.	Identity (%)
	R2A		HM				
	SU	CO	SU	CO			
A1	-	0.6	-	-	*Nocardioides oleivorans*	EU977821	99
A2	2.7	-	-	-	*Nocardioides fulvus*	AY167844	98
A3	0.5	-	-	-	*Nocardioides oleivorans*	AB365060	100
A4	0.5	-	-	-	*Nocardioides plantarum*	NR 036915	98
A5	-	0.6	-	-	*Nocardioides halotolerans*	NR 044234	97
A6	1.1	-	-	-	*Nocardioides lianchengensis*	JX841006	100
A7	1.1	-	-	-	*Aeromicrobium ginsengisoli*	NR 041384	99
A8	2.1	2.5	-	-	*Aeromicrobium ginsengisoli*	NR 041384	100
A9	-	0.6	-	-	*Aeromicrobium panaciterrae*	NR 041382	99
A10	1.1	1.2	-	-	*Nocardioides jensenii*	NR 044981	97
A11	0.5	-	-	-	*Kribbella koreensis*	AY253865	99
A12	0.5	-	-	-	*Mycobacterium peregrinum*	HE575962	100
A13	1.1	-	-	-	*Mycobacterium pyrenivorans*	JX841025	99
A14	2.7	2.5	2.2	5.5	*Mycobacterium sacrum*	GU201853	99
A15	-	-	-	1.1	*Mycobacterium hodleri*	NR 026286	99
A16	-	-	-	1.1	*Nocardia vinacea*	AB162802	100
A17	-	-	1.1	-	*Amycolatopsis balhimycina*	NR 025564	99
A18	-	-	-	1.1	*Leifsonia kafniensis*	NR 042669	98
A19	-	0.6	-	-	*Leifsonia lichenia*	AB278552	100
A20	-	0.6	-	-	*Salinibacterium xinjiangense*	NR 043893	99
A21	-	1.9	-	-	*Agrococcus jejuensis*	HQ896931	100
A22	2.1	3.1	3.3	1.1	*Microbacterium oxydans*	FN908776	100
A23	-	-	1.1	-	*Microbacterium takaoensis*	JX185527	100
A24	-	-	-	1.1	*Microbacterium arborescens*	EU741114	98
A25	-	-	-	1.1	*Pseudoclavibacter helvolus*	GU584997	100
A26	-	0.6	-	-	*Streptomyces cacaoi*	AB184502	99
A27	0.5	-	2.2	-	*Streptomyces rishiriensis*	JQ342919	100
A28	-	0.6	-	-	*Streptomyces turgidiscabies*	HQ441820	100
V1	0.5	-	-	-	*Haloferula helveola*	NR 041673	94
V2	0.5	-	-	-	*Luteolibacter algae*	JX869994	96
V3	1.1	-	-	-	*Verrucomicrobium spinosum*	NR 026266	99

Fig. S1 Continued K. Okazaki
